# Construction of a high-resolution genetic map and identification of quantitative trait loci for salt tolerance in jute (*Corchous* spp.)

**DOI:** 10.1186/s12870-019-2004-7

**Published:** 2019-09-09

**Authors:** Zemao Yang, Youxin Yang, Zhigang Dai, Dongwei Xie, Qing Tang, Chaohua Cheng, Ying Xu, Chan Liu, Canhui Deng, Jiquan Chen, Jianguang Su

**Affiliations:** 1 Institute of Bast Fiber Crops, Chinese Academy of Agricultural Sciences/Key Laboratory of Stem-fiber Biomass and Engineering Microbiology, Ministry of Agriculture, Changsha, 410205 People’s Republic of China; 2 Department of Horticulture, College of Agronomy, Jiangxi Agricultural University, Nanchang, 330045 People’s Republic of China; 3 Institute of Industrial Crops, Heilongjiang Academy of Agricultural Sciences, Harbin, 150000 People’s Republic of China

**Keywords:** Jute, Linkage groups, Quantitative trait loci mapping, Salt tolerance, Genotyping-by sequencing (GBS)

## Abstract

**Background:**

Jute (*Corchorus* spp.) is the most important natural fiber crop after cotton in terms of cultivation area and production. Salt stress greatly restricts plant development and growth. A high-density genetic linkage map is the basis of quantitative trait locus (QTLs) mapping. Several high-density genetic maps and QTLs mapping related to salt tolerance have been developed through next-generation sequencing in many crop species. However, such studies are rare for jute. Only several low-density genetic maps have been constructed and no salt tolerance-related QTL has been mapped in jute to date.

**Results:**

We developed a high-density genetic map with 4839 single nucleotide polymorphism markers spanning 1375.41 cM and an average distance of 0.28 cM between adjacent markers on seven linkage groups (LGs) using an F2 jute population, LGs ranged from LG2 with 299 markers spanning 113.66 cM to LG7 with 1542 markers spanning 350.18 cM. In addition, 99.57% of gaps between adjacent markers were less than 5 cM. Three obvious and 13 minor QTLs involved in salt tolerance were identified on four LGs explaining 0.58–19.61% of the phenotypic variance. The interval length of QTL mapping varied from 1.3 to 20.2 cM. The major QTL, qJST-1, was detected under two salt stress conditions that explained 11.81 and 19.61% of the phenotypic variation, respectively, and peaked at 19.3 cM on LG4.

**Conclusions:**

We developed the first high-density and the most complete genetic map of jute to date using a genotyping-by-sequencing approach. The first QTL mapping related to salt tolerance was also carried out in jute. These results should provide useful resources for marker-assisted selection and transgenic breeding for salt tolerance at the germination stage in jute.

**Electronic supplementary material:**

The online version of this article (10.1186/s12870-019-2004-7) contains supplementary material, which is available to authorized users.

## Background

Jute is a diploid (2n = 14) Malvaceae herb plant that includes 50–60 species [1]. It is the most important and used natural fiber crop in the world after cotton in both the cultivation area and production [2]. *Corchorus capsularis* and *Corchorus olitorius* are the only two cultivated species, with genome sizes of 338 Mb and 445 Mb, respectively [3]. Jute can rapidly and easily grow in nutrient-poor soil while still producing a large amount of biomass. Jute fiber is soft, shiny, easy to dry, shows good hygroscopicity and antibacterial properties, and is degradable, recyclable, and environment friendly [4]. In recent years, the application of jute has been expanding, and it is now often employed in the paper and textile industries and as a herbal medicine, leafy vegetable, and renewable biofuel source [5–8]. Thus, the global demand for jute is increasing [9]. However, very few novel jute breeding approaches have been developed, and progress in breeding and genetic improvement has been slow over the past several decades compared with that for other bast fiber crops [10].

Salt stress is a major environmental factor that limits crop productivity in agriculture and threatens food security [11]. Approximately 20% of all irrigated lands are affected by salinity [12], which is expected to become more serious and expansive in the face of global climate change and environmental pollution [13]. Salt stress causes both primary and secondary effects in a plant. Primary effects include both osmotic and ion-toxicity effects on cells, whereas secondary effects are complex, including oxidative stress, damage to cellular components, and metabolic dysfunction [11]. Therefore, understanding the genetic mechanisms underlying plant salt tolerance and mapping quantitative trait loci (QTLs) related to salt tolerance are crucial for improving the salt tolerance of economically important plants.

A high-density genetic linkage map is the basis of QTLs mapping. Several high-density genetic maps and QTLs mapping related to salt tolerance have been developed using next-generation sequencing in many crop species [14–16]. However, such studies are rare for jute. Nevertheless, several studies related to the construction of genetic maps and identification of QTLs have been reported in this plant. For example, Tao et al. [10] constructed a genetic map with 913 polymorphic markers and detected 11 plant height-related QTLs using 100 F8 lines in jute (*C. capsularis* L.), representing the highest density genetic linkage map of jute reported to date. Kerdu et al. [2] subsequently developed a genetic map including 503 restriction site-associated DNA markers spanning 358.5 cM, with an average marker interval of 0.72 cM that covered 87.0% of the *C. olitorius* L. genome. Nine QTLs were detected for traits correlated with histological fiber content. Biswas et al. [17] developed the third density map for jute, including 48 simple sequence repeats (SSRs) and 410 single nucleotide polymorphisms (SNPs) in *C. olitorius* L. In addition, several low-density genetic linkage maps were constructed using molecular markers based on conventional polymerase chain reaction (PCR) technology such as simple sequence repeats (SSRs), amplified fragment length polymorphisms, and the like, revealing some QTLs associated with fiber yield and quality traits based on genetic linkages [18–23]. However, no QTL related to salt tolerance has been mapped in jute to date.

In this study, we developed the highest density genetic map reported thus far using an F2 population derived from a cross between a wild germplasm of *C. olitorius* L. (J009) and the Guangfengchangguo (GFG) variety. Using the genetic map, we identified major and minor QTLs involved in salt tolerance, and determined the phenotypic variance. These results should provide useful resources for marker-assisted selection and transgenic breeding for salt tolerance at the germination stage in jute.

## Results

### Genotyping by sequencing (GBS)

To enhance the chance of detecting as many SNP markers as possible, the two parents were sequenced at high levels. In total, 16.68 Gb clean bases (average Q20 = 95.30%) were generated with each read of 150 bp for the two parents, GFG and J009, accounting for a total of 8,282,642,100 bp and 8,399,779,200 bp, respectively (see Additional file 1: Table S1)). For the 150 F_2_ individuals, a total of 113.47 Gb clean bases were obtained with an average Q20 value of 95.56%, ranging from 447.55 Mb to 1087.85 Mb and an average length of 756.57 Mb per individual (see Additional file 1: Table S1). The raw data were deposited into the NCBI under accession number PRJNA544984. After aligning the high-quality clean reads against the jute reference genome, an average of 93.12% clean data were mapped covering 97.24% of the reference genome (at least one base covered) with an average coverage depth of 20.97× in the two parents (see Additional file 3: Figure S1). On average, 94.92% of the clean data were aligned in the progenies, covering 21.36% of the jute reference genome with an average 9.71× cover depth (see Additional file 3: Figure S1). Only reads aligned to a unique position on the reference genome were used for SNP calling and genotyping.

### SNP discovery and genotyping

A total of 1,959,620 SNPs were detected in the two parents, which were classified into eight segregation types according to the CP model in JoinMap 4.0 (see Additional file 4: Figure S2). The segregation types hk × hk, nn × np, and aa × bb accounted for 51.93, 27.51, and 11.09% of the detected SNP markers, respectively. Only the marker type aa × bb could be used for the genetic map of the F2 population. According to the marker type (aa × bb) position in the genome, the corresponding genotypes were selected in the 150 progeny plants. In total, 8150 effective markers were used for the genetic mapping, which showed non-significantly distorted segregation, good integrity (> 75%), and no abnormal bases.

### High-resolution genetic map construction

**Fig. 1:**
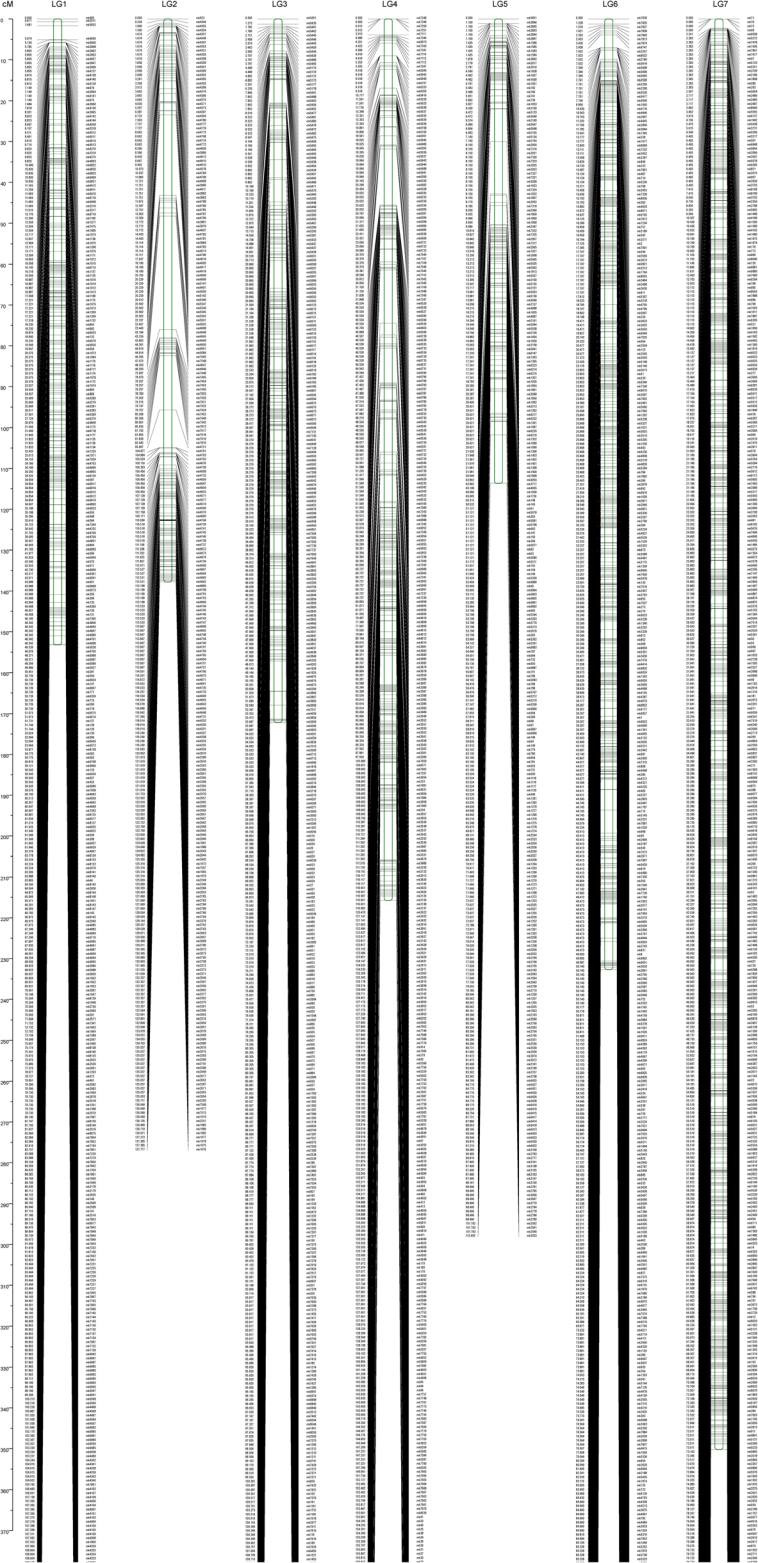
A high-density genetic map in jute comprised 4839 markers on seven linkage groups designated as LG1-LG7

Ultimately, 4839 SNPs of effective markers could be successfully assigned to unique genetic positions (see Additional file 2: Table S2). Therefore, a high-density genetic map was developed comprising 4839 markers on seven linkage groups designated LG1–7 (Fig. 1). The total length of the genetic map was 1375.41 cM with an average distance of 0.28 cM between adjacent markers (Table 1), 99.57% of which with less than 5-cM. Among the seven LGs, LG7 was the largest linkage group, including 1542 markers spanning 350.18 cM with an average distance of 0.23 cM between adjacent markers. The second largest linkage group was LG6 covered by 991 markers spanning 232.68 cM, also with an average distance of 0.23 cM. LG2 was the shortest group with 113.66 cM, including 299 markers, with an average distance of 0.38 cM between markers. LG5 had the least number of markers (278) and the maximum average distance between adjacent markers (0.50 cM).

**Table 1:** statistics on basic characteristics of the seven LGs

Group	Marker number	Total distance (cM)	Average distance (cM)	Gaps < 5 (%)
LG1	517	153.21	0.3	100.00
LG2	278	137.78	0.5	98.56
LG3	635	172.18	0.27	99.37
LG4	577	215.72	0.37	98.79
LG5	299	113.66	0.38	98.66
LG6	991	232.68	0.23	99.80
LG7	1542	350.18	0.23	100.00
total	4839	1375.41	0.28	99.57

Gap < 5: indicating the percentages of gaps in which the distance between adjacent markers was smaller than 5 cM

### Identification of salt tolerance-related QTLs

**Fig. 2:**
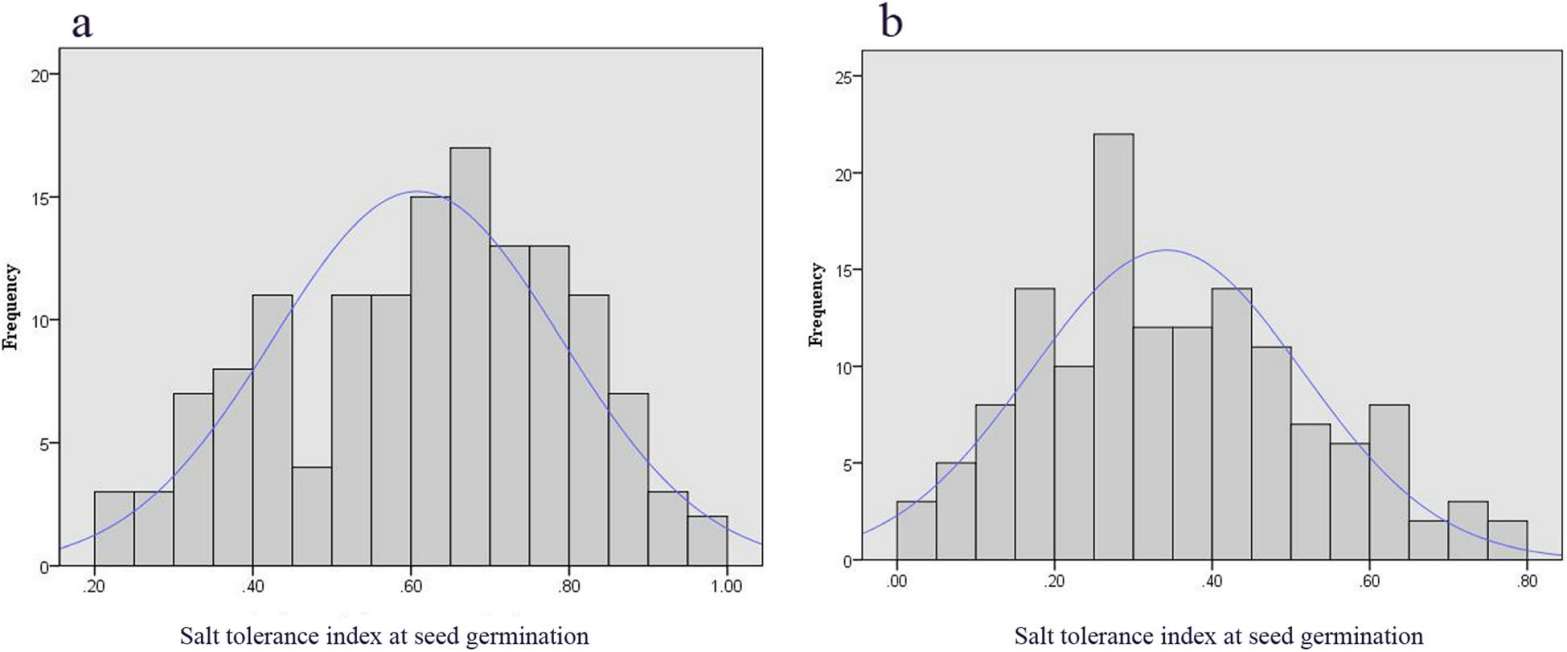
Frequency distribution of salt tolerance index at seed germination of the F_2:3_ population and their parents of jute under 140 mM (**a**) and 160 mM (**b**) on the fourth day underlying salt treatment

**Fig. 3:**
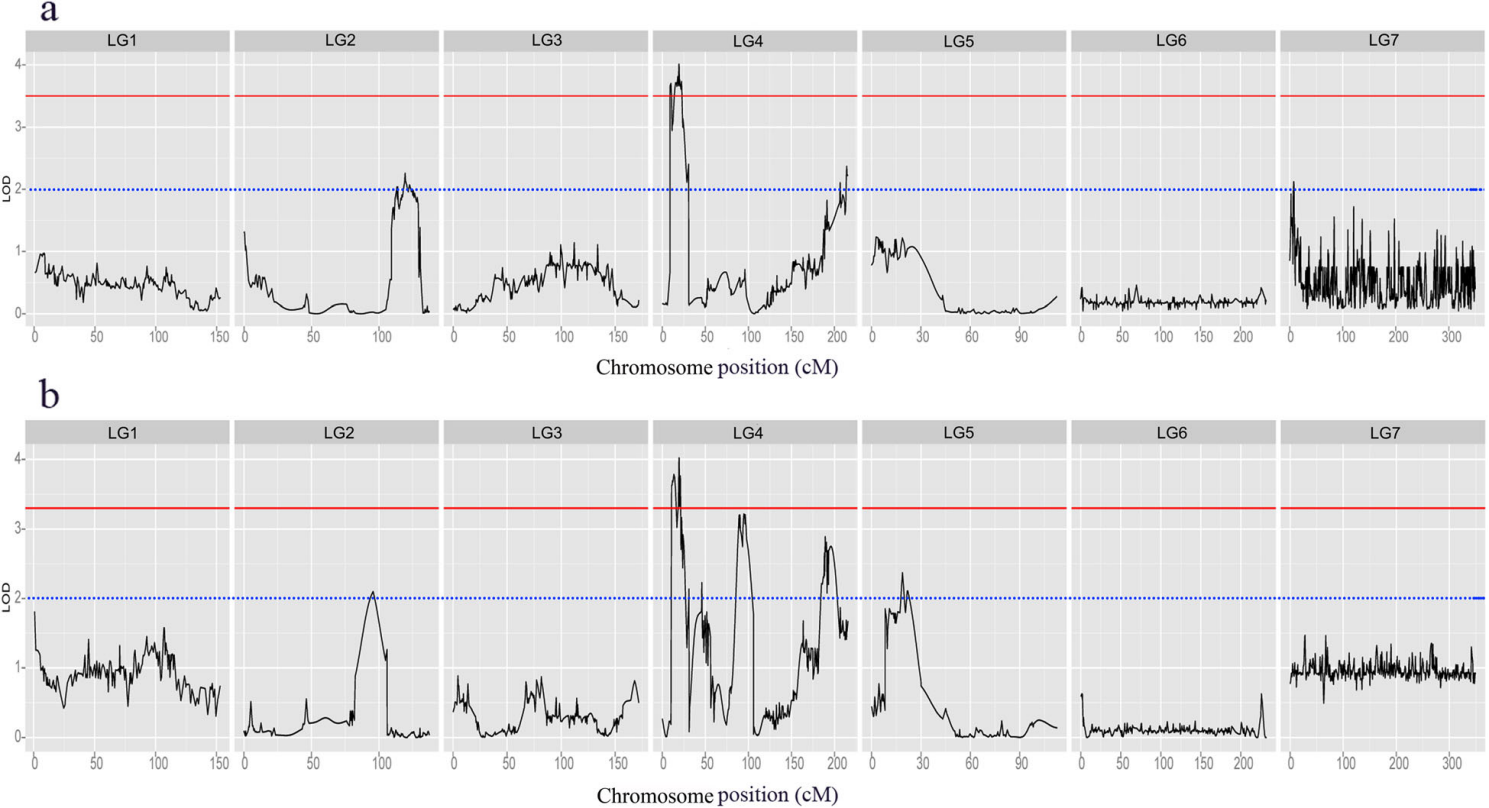
QTL mapping results of salt tolerance in jute under 140 mM (**a**) and 160 mM (**b**)

We recorded the salt tolerance index at seed germination (STIG) from the 150 F_2:3_ lines and two parents under two salt stress conditions for six days. The STIG data on the fourth day exhibited a normal distribution (Fig. 2), and were used as the phenotype data of QTLs. According to the 1000-permutations test, the LOD score threshold was determined to be about 3.5 in the two salt stress conditions. We identified three obvious QTLs with over 3.5 LOD values on LG4 under the two salt stress conditions, and a major QTL was detected under the two conditions simultaneously, designated qJST-1 (Table 2; Fig. 3). The QTL qJST-1 was located at 11.4–23.7 cM between markers mk5633 and mk6723 on LG4 under the 140 mM salt stress condition and at 16.9–21.6 cM between markers mk6160 and mk6484 on LG4 under the 160 mM salt stress condition. The LOD peak of qJST-1 mapped at 19.31 cM on LG4 under both the 140 mM and 160 mM salt stress conditions, explaining 11.81 and 19.61% of the total phenotypic variation, respectively. The QTL qJST-2 was located between markers mk7047 and mk5638 (at the 9–11.4 cM position of LG4) under the 140 mM salt stress condition, and the LOD peak mapped at 10.01 cM on LG4, explaining 3.74% of the phenotypic variation. The QTL qJST-3 was detected under the 160 mM salt stress condition, located between markers mk6393 and mk6391 (10.4–16.9 cM) on LG4 partially overlapping with qJST-2. The LOD peak of qJST-3 mapped at 13.41 cM on LG4, explaining 8.84% of the phenotypic variation. All of the additive effects for the QTLs were negative, indicating that the increased salt tolerance values were conferred by J009 alleles. In addition, although the LOD values were below 3.5 at several positions, the obvious peaks (LOD > 2.0) were discovered in these positions, which are considered to be the regions involved in slightly effective (i.e., minor) QTLs. A total of 13 minor QTLs were identified under the 140 mM (*n* = 5) and 160 mM (*n* = 8) salt stress condition (Table 2, Fig. 3), which mapped to LG2, LG4, LG5 and LG7, and explained 0.58–8.12% of the phenotypic variance. The interval length of QTL mapping varied from 1.3 to 20.2 cM.

**Table 2:** QTLs related to salt tolerance of jute in the two salt stress conditions

QTLs name	salt stress (mM)	LGs	location interval (cM)	Peak Position (cM)	Left Marker	Right Marker	LOD	Additive_effect	PVE(%)
qJST-1	140	LG4	11.4–23.7	19.31	mk5633	mk6723	4.01	− 0.24	11.81
	160	LG4	16.9–21.6	19.31	mk6160	mk6484	4.02	−0.22	19.61
qJST-2	140	LG4	9–11.4	10.01	mk7047	mk5638	3.70	−0.08	3.74
qJST-3	160	LG4	10.4–16.9	13.41	mk6393	mk6391	3.78	−0.11	8.84
qJST-4	140	LG2	111.3–113.5	113.31	mk4674	mk4906	2.04	0.06	5.30
qJST-5	140	LG2	114.9–121.6	119.31	mk4671	mk3289	2.26	0.07	4.52
qJST-6	140	LG4	193.9–207.5	206.71	mk3488	mk7719	2.11	−0.02	1.40
qJST-7	140	LG4	212.3–214.7	214.31	mk7722	mk7984	2.37	−0.01	1.91
qJST-8	140	LG7	6.4–8.7	7.41	mk5217	mk2356	2.12	−0.24	0.70
qJST-9	160	lg02	84.7–104.9	95.71	mk4721	mk4763	2.10	0.05	6.43
qJST-10	160	lg04	35.8–46.5	45.91	mk6526	mk6728	2.23	0.07	0.58
qJST-11	160	lg04	93.3–96.0	94.61	mk3387	mk3553	3.22	−0.08	6.36
qJST-12	160	lg04	96–103.3	96.31	mk3561	mk7900	3.21	−0.08	5.51
qJST-13	160	lg04	188.6–189.9	189.31	mk2761	mk3020	2.89	0.07	7.58
qJST-14	160	lg04	191.3–192.9	192.41	mk3017	mk3379	2.69	0.06	5.90
qJST-15	160	lg05	14.9–20.6	18.81	mk2158	mk1092	2.37	0.05	8.12
qJST-16	160	lg05	20.6–22.0	22.0	mk6213	mk1400	2.11	0.05	7.17

PVE: explaining percentage of the phenotypic variatiation

## Discussion

### Construction of the highest density genetic map of jute to date

Although several genetic maps for jute have been constructed with QTLs identified for economically important traits, most of this work involved the use of traditional DNA markers such as amplified fragment length polymorphisms, random amplified polymorphic DNA, and inter-simple sequence repeats [18–23]]. Generation of such markers is labor-intensive, expensive, and of low-throughput, and thus not suitable for the construction of a high-resolution genetic map and fine QTLs mapping [10]. The development of next-generation sequencing has provided an automated, cost-effective, and high-throughput method for identifying SNP markers, which is also conducive to constructing high-density genetic maps and fine QTL mapping. However, only a few studies [2, 10, 17] have incorporated the markers developed by high-throughput technology for constructing jute genetic and QTL maps. To our knowledge, the densest genetic map for jute constructed to date was developed using specific locus amplified fragment sequencing, including only 913 markers in 11 LGs covering 1621.4 cM with an average density of 1.61 cM per locus [10]. In the present study, we developed the first high-density and the most complete genetic map of jute using a GBS approach. The genetic map comprised 4839 markers on seven LGs, consistent with the number of chromosomes in jute (*n* = 7), spanning 1375.41 cM with an average distance of 0.28 cM per locus, which is lower than that reported previously. This genetic map will thus greatly enhance QTL/gene mapping and marker-assisted breeding in jute.

### Phenotyping of salt tolerance in jute

Salt tolerance is a quantitative genetic characteristic controlled by multiple genes in many crops [15]. Accurate phenotyping is an important part of QTLs mapping [24, 25]. Thus, we measured the relative seed germination percentage between salt-tolerant and control lines under two salt stress conditions over six days with three biological replicates. The germination rates of all F_2:3_ lines were very low over the first two days under salt stress conditions. In the last two days, the phenotype of salt tolerance polarized to a skewed distribution, then was mostly consistent with a normal distribution on the fourth day. Accordingly, we chose the phenotyping data of salt tolerance on the fourth day for QTL mapping.

### QTL mapping of salt tolerance in jute

Salt stress is a major environmental stressor that restricts plant development and limits the yield of crop plants [26]. Crop salt tolerance can be enhanced by precise breeding, which requires the identification of salt tolerance-related genes or molecular markers. However, most previous studies of salt tolerance in jute have focused only on the morphological, physiological, and proteomic components [26]. To our knowledge, only two studies [9, 27] related to the salt tolerance of jute have been carried out through transcriptome sequencing, revealing a large number of differentially expressed genes underlying salt tolerance. However, the large number of differentially expressed genes made it difficult to identify the best candidate genes related to salt tolerance for practical application.

Thus, we carried out QTLs mapping to locate candidate genes over a limited interval of the genome. We successfully located 16 QTLs related to salt tolerance. Interestingly, three obvious QTLs, including a major QTL, and six minor QTLs were discovered within LG4. These findings indicate that this LG might play a crucial role in the stress resistance of jute. All additive effects were negative for the three obvious QTLs, suggesting that the increased trait values are conferred by J009 alleles.

Overall, these results highlight the presence of excellent resistance genes in wild jute plants. The utilization of salt tolerant wild germplasm resources is conducive to accelerating the cultivation of resistant jute varieties. The major QTL, qJST-1, was detected under the two salt stress conditions, and the location interval (16.9–21.6 cM) in the 160 mM salt stress condition was also included in that detected in the 140 mM condition. Therefore, this position may harbor the salt tolerance genes in jute. Thus, we plan to focus on the genes in this region in a future work to further identify candidate genes for salt tolerance. Cloning these genes could have potential applications in both marker-assisted selection and transgenic breeding for salt tolerance in crops.

## Conclusions

We developed the first high-density and the most complete genetic map of jute to date using a GBS approach. Further, the first QTL mapping related to salt tolerance also was carried out in jute. Three obvious and 13 minor QTLs involved in salt tolerance were identified on four LGs. The major QTL, qJST-1, was detected under two salt stress conditions that explained 11.81 and 19.61% of the phenotypic variation, respectively. These results should provide useful resources for marker-assisted selection and transgenic breeding for salt tolerance at the germination stage in jute. However, the QTLs could not be mapped to an interval on chromsomes, because the jute reference genome is only a scaffold version not anchored to chromosomes. Therefore, the genes contained in these QTL intervals could not be identified. In addition, the F_2:3_ were used for QTL mapping in the study. Which is more suitable for QLT preliminary mapping than for fine mapping. In the future, recombinant inbred lines and a high-quality reference genome of jute will be developed and constructed, which will be of great value for cloning these salt-tolerant genes and applying them to jute salt-tolerant breeding.

## Methods

### Mapping population and DNA extraction

The two parent plants used in this study were the wild *C. olitorius* L. germplasm J009 and the *C. olitorius* L variety Guangfengchangguo (GFG). They originally came from Nepal and Guangfeng, Jiangxi Province, China, respectively. And J009 was more salt tolerant than GFG. Both were stored in the National Bast Fiber Germplasm Middle-term Storage of China (in Changsha, Hunan province, China). The F2 population consisting of 150 plants and their F2:3 progeny were used for the construction of a high-resolution genetic map and QTLs mapping under salt stress. Young leaf tissues from the 150 F2 individuals and two parents were collected for genomic DNA extraction. The total genomic DNA per individual was extracted using the DNeasy Plant Mini Kit (TIANGEN, Beijing, China), and used in genotyping-by sequencing (GBS) library preparation for SNP discovery. DNA degradation and contamination was monitored on 1% agarose gels. DNA purity was checked using a NanoPhotometer® spectrophotometer (IMPLEN, CA, USA), and the DNA concentration was measured using Qubit® DNA Assay Kit with the Qubit® 2.0 flurometer (Life Technologies, CA, USA).

### Evaluation of salt tolerance at the germination stage

The salt tolerance experiment was carried out under two salt concentration conditions, 140 mM and 160 mM, both with a 12 h–12 h day/night photoperiod, corresponding to a temperature of 28 ± 0.5 °C/25 ± 0.5 °C and relative humidity of 75% in an illumination incubator. Surface sterile seeds of the mapping population (150 F_2:3_ lines) were sown onto clean and sterile petri dishes with two uniform sterile filter papers. Each line was sown on two petri dishes with 30 seeds per dish. One dish was used as the salt stress condition and the other served as the control. For salt stress induction, 3 ml of NaCl solution and 3 ml water was applied to the salt stress and control dishes, respectively, on the first day, and then 2 ml of NaCl solution and 2 ml water were added to each corresponding plate for six days. A randomized complete design with three biological replicates was adopted in each salt stress condition. The condition of seed germination was observed and recorded every day throughout the six-day experiment. The seeds were defined as germinated seeds when the buds were longer than 3 mm. The STIG was evaluated according to the following equation:

$$ \mathrm{STIG}=\frac{the\ number\ of\ germinated\ seeds\ under\ salt\ stress}{the\ number\ of\ seeds}/\frac{the\ number\ of\ germinated\ seeds\ under\ control\ condition}{the\ number\ of\ seeds} $$

### GBS library construction and sequencing

The GBS library was constructed according to standard GBS protocol [28, 29]. DNA of each individual of the F2 population and parents was prepared by restriction digestion with MseI and HaeIII, followed by ligation with barcoded adapters for individual labeling. The restriction ligation samples with different barcode sequences were purified with Agencourt AMPure XP (Beckman). PCR was then performed using purified samples with Phusion Master Mix (NEB) universal primer and index primers for amplification of the complete i5 and i7 sequences. The PCR products were purified using Agencourt AMPure XP (Beckman) and pooled, and then run on a 2% agarose gel. Fragments of 375–400 bp (including indexes and adaptors) were isolated using a gel extraction kit (Qiagen). These fragment products were then purified using Agencourt AMPure XP (Beckman), and used for pair-end sequencing with the Illumina HiSeq™ 2500 platform.

### SNP calling and genotyping

The sequences of each sample were sorted according to individual barcodes, and the raw data were processed through a series of quality control procedures based on in-house C scripts according to the standards described by Nie et al. [30] . Burrows-Wheeler Aligner [31] was used to align the clean reads of each sample against the reference genome (settings: mem -t 4 -k 32 –M -R). Alignment files were converted to BAM files using SAMtools software [32] (settings: –bS –t). If multiple read pairs showed identical external coordinates, only the read pairs with the highest mapping quality were retained. SNP calling was performed for all samples using GATK [33] software and filtered with a perl script.

### Genetic map construction

Polymorphic markers of the parents were classified into eight segregation patterns (ab × cd, ef × eg, hk × hk, lm × ll, nn × np, aa × bb, ab × cc, and cc × ab). Markers showing significantly distorted segregation (*P* < 0.001), integrity (< 75%), or containing abnormal bases were filtered using JoinMap 4.0 [34]. The aa × bb segregation pattern was used for constructing the genetic map for the F2 population. JoinMap 4.0 software was used to sort the markers in every linkage group. The Kosambi mapping function was used for marker distance calculation. The logarithm of odds (LOD) value was set to 2.0–30. A Perl script SVG was used to draw the linkage maps and construct heat maps for evaluation.

### QTL mapping for salt tolerance

The genotyping data of the mapped SNPs on the seven linkage maps were integrated with the salt tolerance phenotype data per plant for QTL mapping. The QTL analysis was performed using QTL Cartographer v2.5 [35] based on the composite interval mapping method (CIM). For each trait, the LOD score threshold was determined by a 1000-permutation test at all markers with a significance level of *P* < 0.05 using MapQTL [36]. The mapped interval of each QTL was determined based on its LOD peak location and the surrounding region over the score threshold.

## Additional files

Additional file 1: **Table S1.** Sequencing results information statistics (XLSX 8 kb)

Additional file 2: **Table S2.** Information of 4839 SNPs of effective markers successfully assigned to unique genetic positions (XLSX 3232 kb)

Additional file 3: **Figure S1.** Sequencing information for each of the F2 individuals and their parents. Average coverage depth and percentage of coverage jute reference genome of each of the F2 individuals and their parents was displayed in a and b, respectively. (JPG 709 kb)

Additional file 4: **Figure S2.** Number of markers for eight segregation patterns (JPG 191 kb)

## Data Availability

All data generated or analysed during this study is contained within the manuscript and additional files. And the all of raw data used in our study have been deposited into the NCBI under accession number PRJNA544984.

## Abbreviations

CIM: Composite interval mapping method
GBS: Genotyping-by sequencing
LGs: Linkage groups
PCR: Polymerase chain reaction
QTL: Quantitative trait locus
SNP: Single nucleotide polymorphism
SSRs: Simple sequence repeats
STIG: Salt tolerance index at seed germination

## References

[1] Yang Z, Lu R, Dai Z, Yan A, Chen J, Bai Z, Xie D, Tang Q, Cheng C, Xu Y. Analysis of genetic diversity and population structure of a worldwide collection of *Corchorus olitorius L.* germplasm using microsatellite markers. Biotechnol Biotec Eq. 2018:1–7.

[2] Kundu A, Chakraborty A, Mandal NA, Das D, Karmakar PG, Singh NK, Sarkar D (2015). A restriction-site-associated DNA (RAD) linkage map, comparative genomics and identification of QTL for histological fibre content coincident with those for retted bast fibre yield and its major components in jute (*Corchorus olitorius L., Malvaceae s. l.*). Molecular Breeding.

[3] Islam MS, Saito JA, Emdad EM, Ahmed B, Islam MM, Halim A, Hossen QMM, Hossain MZ, Ahmed R, Hossain MS (2017). Comparative genomics of two jute species and insight into fibre biogenesis. Nature Plants.

[4] Islam MK, Alam I, Khanam MS, Siyoung L, Waghmode TR, Mooryong H (2014). Accumulation and tolerance characteristics of chromium in nine jute varieties (*Corchorus spp.* and *Hibiscus spp.*). Plant Omics.

[5] Dansi A, Adjatin A, Adoukonousagbadja H, Faladé V, Yedomonhan H, Odou D, Dossou B (2008). Traditional leafy vegetables and their use in the Benin Republic. Genetic Resources and Crop Evolution.

[6] Zeghichi S, Kallithraka S, Simopoulos AP (2003). Nutritional composition of molokhia (*Corchorus olitorius*) and stamnagathi (*Cichorium spinosum*). World Rev Nutr Diet.

[7] Wazni MW, Islam AS, Taliaferro JM, Anwar N, Sathasivan K (2009). Novel ESTs from a jute (*Corchorus olitorius L.*) cDNA library. Plant Tissue Cult Biotechnol.

[8] Islam MM (2013). Biochemistry, medicinal and food values of jute (*Corchorus capsularis L.* and *C. olitorius L.*) leaf: a review. International Journal of Enhanced Research in Science Technology and Engineering.

[9] Yang Z, Lu R, Dai Z, Yan A, Tang Q, Cheng C, Xu Y, Yang W, Su J (2017). Salt-stress response mechanisms using de novo transcriptome sequencing of salt-tolerant and sensitive Corchorus spp. Genotypes Genes.

[10] Tao A, Wu G, Qi J, Xu J, Fang P, Lin L, Zhang L, Lin P (2017). High-density genetic map construction and QTLs identification for plant height in white jute (*Corchorus capsularis L.*) using specific locus amplified fragment (SLAF) sequencing. Bmc Genomics.

[11] Zhu JK (2016). Abiotic stress signaling and responses in plants. Cell..

[12] Al-Tamimi N, Brien C, Oakey H, Berger B, Saade S, Ho YS, Schmöckel SM, Tester M, Negrão S (2016). Salinity tolerance loci revealed in rice using high-throughput non-invasive phenotyping. Nat Commun.

[13] Fedoroff NV, Battisti DS, Beachy RN, Cooper PJ, Fischhoff DA, Hodges CN, Knauf VC, Lobell D, Mazur BJ, Molden D (2010). Radically rethinking agriculture for the 21st century. Science..

[14] Diouf L, Pan Z, He SP, Gong WF, Jia YH, Magwanga RO, Romy K, Or HR, Kirungu JN, Du X (2017). High-density linkage map construction and mapping of salt-tolerant QTLs at seedling stage in upland cotton using genotyping by sequencing (GBS). Int J Mol Sci.

[15] Luo M, Zhao Y, Zhang R, Xing J, Duan M, Li J, Wang N, Wang W, Zhang S, Chen Z (2017). Mapping of a major QTL for salt tolerance of mature field-grown maize plants based on SNP markers. BMC Plant Biol.

[16] Patil G, Do T, Vuong TD, Valliyodan B, Lee JD, Chaudhary J, Shannon JG, Nguyen HT. Genomic-assisted haplotype analysis and the development of high-throughput SNP markers for salinity tolerance in soybean. Scientific Reports. 2016;6(19199). 10.1038/srep19199 PMC4726057 26781337

[17] Biswas C, Dey P, Karmakar PG, Satpathy S (2015). Discovery of large-scale SNP markers and construction of linkage map in a RIL population of jute ( *Corchorus capsularis* ). Mol Breed.

[18] Chen Y, Zhang L, Qi J, Chen H, Tao A, Xu J, Lin L, Fan P (2015). Genetic linkage map construction for white jute (*Corchorus capsularis L.*) using SRAP, ISSR and RAPD markers. Plant Breed.

[19] Topdar N, Kundu A, Sinha MK, Sarkar D, Das M, Banerjee S, Kar CS, Satya P, Balyan HS, Mahapatra BS (2013). A complete genetic linkage map and QTL analyses for bast fibre quality traits, yield and yield components in jute (*Corchorus olitorius L.*). Tsitol Genet.

[20] Das M, Banerjee S, Topdar N, Kundu A, Mir RR, Sarkar D, Sinha MK, Balyan HS, Gupta PK (2012). QTL identification for molecular breeding of fibre yield and fibre quality traits in jute. Euphytica..

[21] Zhang L, Liu X, Zhang L, Wan X, Tao A, Fang P, Lin P, Qi J. Construction of a genetic map using newly developed SSR markers for identifying QTL for plant height in jute ( *Corchorus capsularis* ). Crop J. 2016.

[22] Sultana N, Khan H, Ashraf N, Sharkar MTK (2006). Construction of an intraspecific linkage map of jute. Asian J Plant Sci.

[23] Haque S, Ashraf N, Begum S, Sarkar RH, Khan H (2009). Construction of genetic map of jute (*Corchorus olitorius L.*) based on RAPD markers. Plant Tissue Cult Biotechnology..

[24] Shi Y, Gao L, Wu Z, Zhang X, Wang M, Zhang C, Zhang F, Zhou Y, Li Z (2017). Genome-wide association study of salt tolerance at the seed germination stage in rice. BMC Plant Biol.

[25] Yang Z, Huang D, Tang W, Zheng Y, Liang K, Cutler AJ, Wu W (2013). Mapping of quantitative trait loci underlying cold tolerance in rice seedlings via high-throughput sequencing of pooled extremes. PLoS One.

[26] Ma H, Yang R, Song L, Yang Y, Wang Q, Wang Z, Ren C (2015). Differential proteomic analysis of salt stress response in jute (*Corchorus capsularis* & *olitorius L.*) seedling roots. Pak J Bot.

[27] Yang Z, Yan A, Lu R, Dai Z, Tang Q, Cheng C, Xu Y, Su J (2017). De novo transcriptome sequencing of two cultivated jute species under salinity stress. PLoS One.

[28] Elshire RJ, Glaubitz JC, Sun Q, Poland JA, Kawamoto K, Buckler ES, Mitchell SE (2011). A robust, simple genotyping-by-sequencing (GBS) approach for high diversity species. PLoS One.

[29] Sonah H, Bastien M, Iquira E, Tardivel A, Légaré G, Boyle B, Normandeau É, Laroche J, Larose S, Jean M (2013). An improved genotyping by sequencing (GBS) approach offering increased versatility and efficiency of SNP discovery and genotyping. PLoS One.

[30] Nie H, Yan X, Huo Z, Jiang L, Chen P, Liu H, Ding J, Yang F (2017). Construction of a high-density genetic map and quantitative trait locus mapping in the Manila clam Ruditapes philippinarum. Sci Rep.

[31] Li H, Durbin R (2009). Fast and accurate short read alignment with burrows–wheeler transform: Oxford University press.

[32] Li H, Handsaker B, Wysoker A, Fennell T, Ruan J, Homer N, Marth G, Abecasis G, Durbin R (2009). The sequence alignment/map format and SAMtools. Bioinformatics..

[33] Auwera GAVD, Carneiro MO, Hartl C, Poplin R, Angel GD, Levymoonshine A, Jordan T, Shakir K, Roazen D, Thibault J (2013). From FastQ data to high confidence variant calls: the GenomeAnalysis toolkit best practices pipeline. Curr Protoc Bioinformatics.

[34] Stam P (2010). Construction of integrated genetic linkage maps by means of a new computer package: join map. Plant J.

[35] Wang S. Windows QTL cartographer 2.5. 2007.

[36] Ooijen JWV, Maliepaard C (2000). MapQTL (tm) version 3.0: software for the calculation of QTL positions on genetic maps. Order-a Journal on the Theory of Ordered Sets & Its Applications.

