# Supplementary figures and images for: Construction of a high-resolution genetic map and identification of quantitative trait loci for salt tolerance in jute (Corchous spp.)
Source: BMC Plant Biol. 2019 Sep 9;19:391. doi: 10.1186/s12870-019-2004-7 (PMC6734509; doi:10.1186/s12870-019-2004-7)

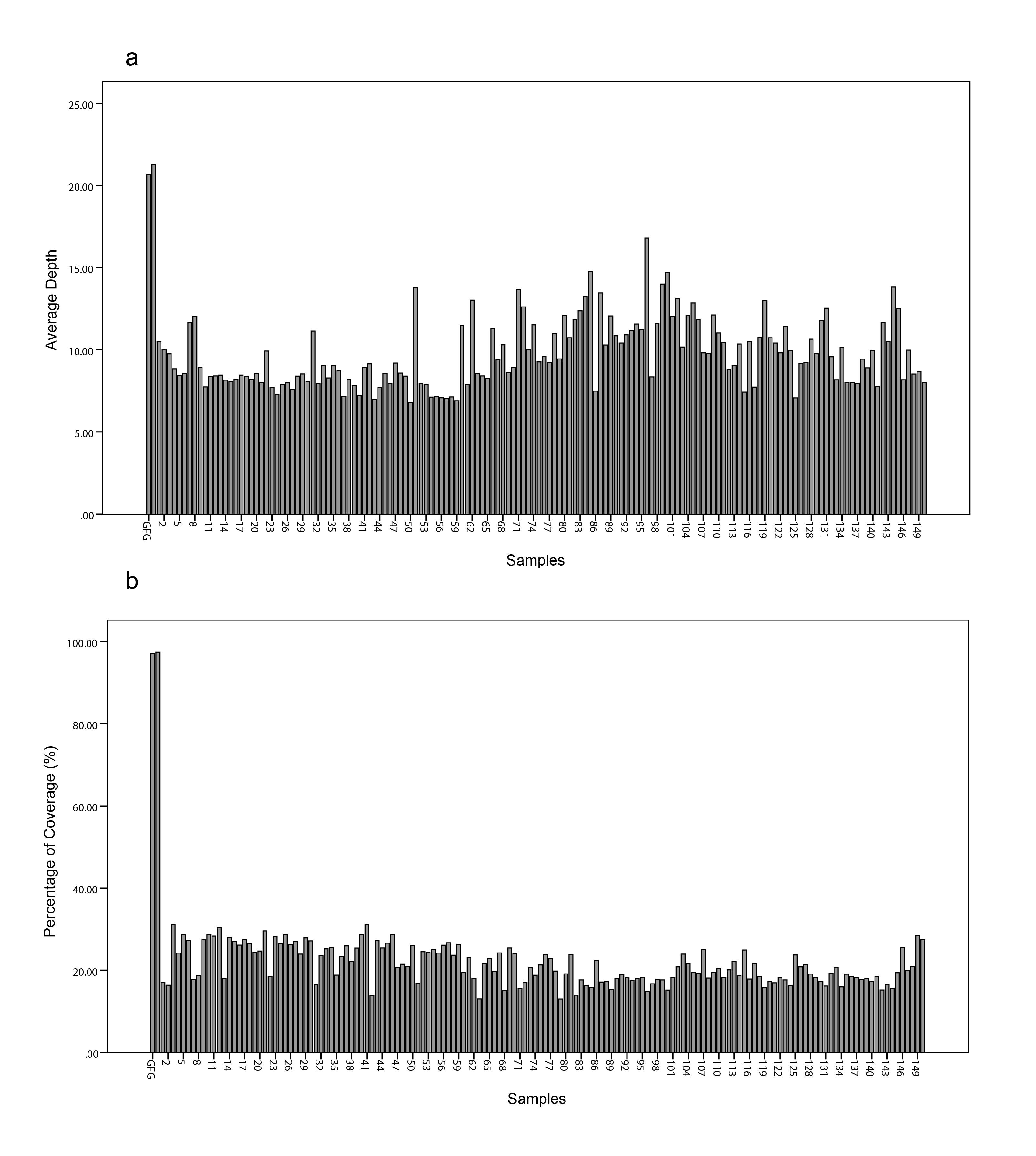

Supplement: Supplementary file 3 — Figure S1. Sequencing information for each of the F2 individuals and their parents. Average coverage depth and percentage of coverage jute reference genome of each of the F2 individuals and their parents was displayed in a and b, respectively. (JPG 709 kb) [file 12870_2019_2004_MOESM3_ESM.jpg]

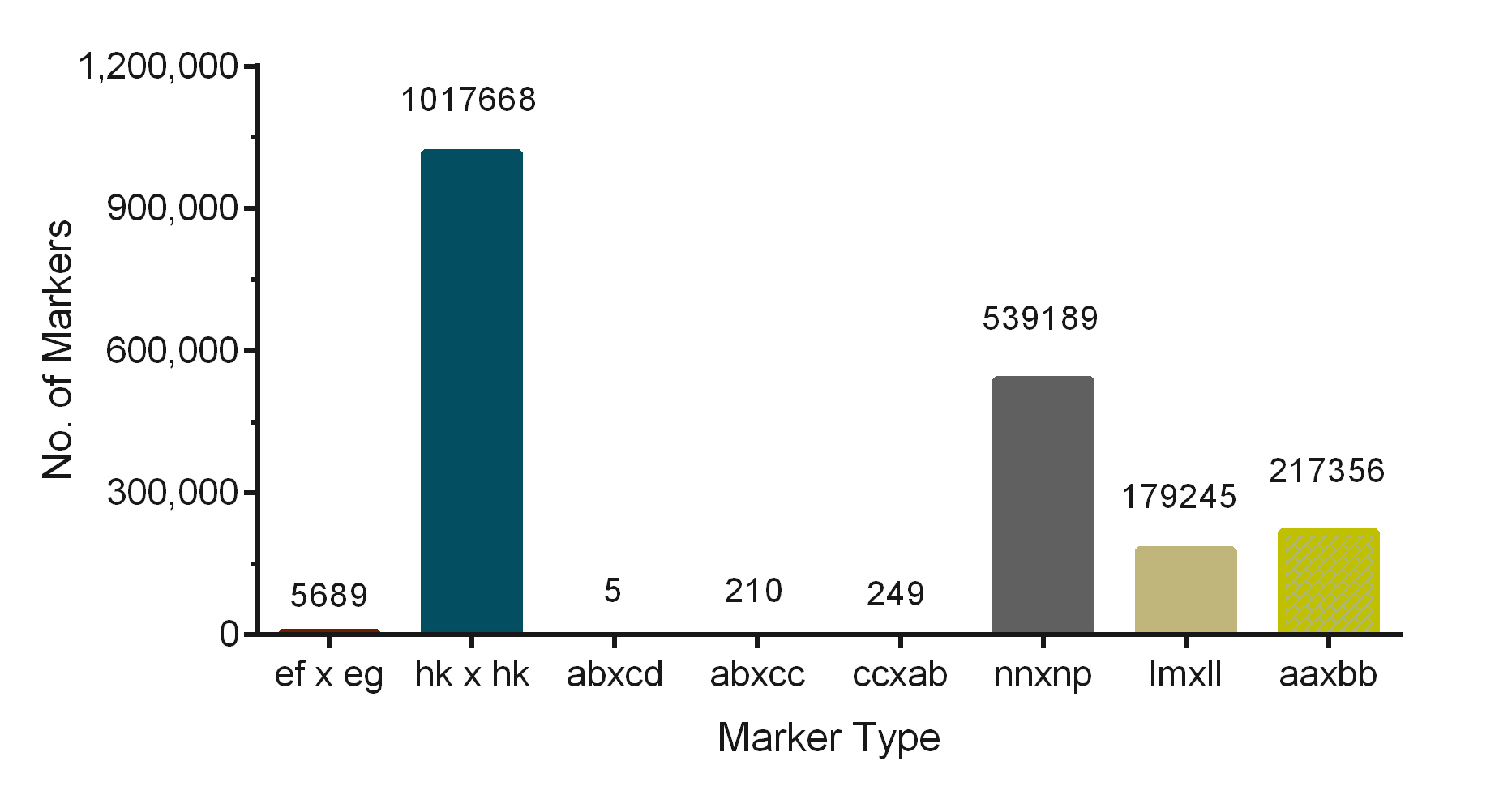

Supplement: Supplementary file 4 — Figure S2. Number of markers for eight segregation patterns (JPG 191 kb) [file 12870_2019_2004_MOESM4_ESM.jpg]
